# Advantages and Prospective Implications of Smart Materials in Tissue Engineering: Piezoelectric, Shape Memory, and Hydrogels

**DOI:** 10.3390/pharmaceutics15092356

**Published:** 2023-09-20

**Authors:** Keisheni Ganeson, Cindy Tan Xue May, Amirul Al Ashraf Abdullah, Seeram Ramakrishna, Sevakumaran Vigneswari

**Affiliations:** 1 Institute of Climate Adaptation and Marine Biotechnolgy (ICAMB), Kuala Nerus 21030, Terengganu, Malaysia; gkeisheni@yahoo.com; 2 Faculty of Science and Marine Environment, Universiti Malaysia Terengganu, Kuala Nerus 21030, Terengganu, Malaysia; s55348@ocean.umt.edu.my; 3 School of Biological Sciences, Universiti Sains Malaysia, Bayan Lepas 11800, Penang, Malaysia; amirul@usm.my; 4 Malaysian Institute of Pharmaceuticals and Nutraceuticals, National Institutes of Biotechnology Malaysia, Gelugor 11700, Penang, Malaysia; 5 Centre for Chemical Biology, Universiti Sains Malaysia, Bayan Lepas 11800, Penang, Malaysia; 6 Center for Nanofibers and Nanotechnology, Department of Mechanical Engineering, National University of Singapore, Singapore 117581, Singapore

**Keywords:** piezoelectric material, polymeric hydrogels, shape-memory material, smart material, stimuli-responsive material, tissue engineering

## Abstract

Conventional biomaterial is frequently used in the biomedical sector for various therapies, imaging, treatment, and theranostic functions. However, their properties are fixed to meet certain applications. Smart materials respond in a controllable and reversible way, modifying some of their properties because of external stimuli. However, protein-based smart materials allow modular protein domains with different functionalities and responsive behaviours to be easily combined. Wherein, these “smart” behaviours can be tuned by amino acid identity and sequence. This review aims to give an insight into the design of smart materials, mainly protein-based piezoelectric materials, shape-memory materials, and hydrogels, as well as highlight the current progress and challenges of protein-based smart materials in tissue engineering. These materials have demonstrated outstanding regeneration of neural, skin, cartilage, bone, and cardiac tissues with great stimuli-responsive properties, biocompatibility, biodegradability, and biofunctionality.

## 1. Introduction

Smart material has been widely used with gradually increasing demand due to the era’s evolutionary development [1]. Smart material is considered a material that can change its physical or functional properties in response to external stimuli or environmental changes [2]; thus, it is also called intelligent or responsive material. Smart material is used in various areas such as automotive, industrial, civil engineering, aerospace, and biomedical. Recently, smart materials have also been contributing significantly to the biomedical sector especially due to their outstanding applications in therapies, imaging, treatment, and theranostic functions [3]. As technology is blooming at its peak, it is crucial to study the prospects of smart materials to improve the healthcare sector.

Although biomaterials such as scaffolds provide mechanical, structural, and physical properties for tissue development, they still do not interact with body cells for functional tissue generation. The human body is constructed with various physiological and pathological sites, where each tissue or organ shows different properties like pH, temperature, and reactive oxygen species. However, a conventional biomaterial exhibits fixed properties that are only customised for certain purposes or applications. It does not adapt to living tissues and undergo changes affected by environmental or biological stimuli. A conventional biomaterial has the concept of one-size-fits-all, therefore escalating the foreign body rejection or reactions in the human body. Therefore, stimuli-responsive smart materials which can have tuneable properties can provide the opportunity to design personalised biomedical products to overcome the limitations of foreign body reactions. It is required for the better performance of any type of tissue engineering. Smart materials can mimic the cell coefficient and behave like natural body tissues. Consequently, smart materials help to speed up the engineering work incorporated with natural tissue and can sustain its effect upon certain stimulation.

Smart materials are generally categorised into two: active and passive smart materials. Active smart materials can transduce force by modifying their properties under certain stimulation [4]. As an example, piezoelectric materials convert electric signals into mechanical force to induce physical deformation via mechanical pressure or vice versa [5]. Material that lacks the inherent capability to transduce energy is considered a passive smart material [4]. Passive smart materials can sense external stimuli but cannot transduce or react; therefore, they are more suitable to be used as sensors like optic fibres. Examples of active smart materials are electrochromic materials, magnetostrictive materials, pyroelectric, shape-memory materials, and piezoelectric [6]. However, this review only focuses on active materials, piezoelectric materials, shape-memory materials, and polymeric hydrogels.

The selection of materials is developing in a more advanced way to meet biomedical needs and is now the future of intelligent materials [1]. Traditionally, smart materials are fabricated using inorganic compounds, metal alloys, and polymers. However, their harsh working conditions and/or high operating voltages limit their practical applications. Smart materials made of polymers are lightweight and have good elasticity as well as high transparency but they also have several disadvantages such as weak mechanical strength, a long response time, and terrible environmental stability [7]. Among natural polymers, proteins are considered extraordinary and flexible building blocks for organisms with complex topological structures and wide-ranging functions [7].

**Figure 1:**
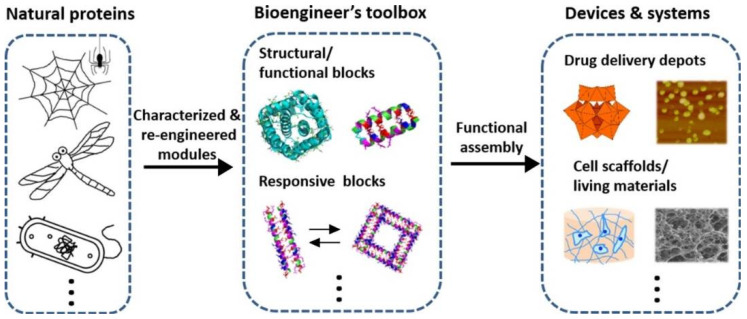
Schematic illustration of how proteins can be bioengineered to be used as smart materials for tissue engineering (Adapted with permission from [9] copyright 2020 Elsevier. Ltd.).

Proteins are excellent candidates for use in smart materials especially due to their programmable self-assembly features which are very promising for various complex structures and functions that can interact specifically and dynamically or result in elaborate systems [7]. Proteins including antibodies, enzymes, and structural proteins such as collagen are abundantly found in nature and they are powerful building blocks that could maintain cell shape and function, promote cell development, and many more [7,8,9]. The use of protein in smart materials enhances its biocompatibility and increases the range of their applications. Proteins also consist of long amino acid sequences with complex 3D structures and usually perform highly specialised tasks in cells [9]. Thus, the function of peptides and proteins to yield self-assembling architectures with optimum physicochemical properties is a significant feature of their application in tissue engineering (Figure 1).

There are also various smart materials fabricated using synthetic materials. This review provides an understanding of the concept of stimuli-responsive smart materials based on proteins on the application, especially in tissue engineering. The advancement and limitations of a protein-based smart material compared to a conventional biomaterial will also be explored.

## 2. Protein-Based Smart Materials in Tissue Engineering

The advancement of biomaterials can be categorised into four levels of “smartness”. A basic biomaterial is considered “inert”, followed by “active”, in which the material will release a certain compound [10]. The third level is “responsive”, in which the material can sense the environmental changes and react to a specific biofunction. The highest level of material will be “autonomous”, which the material can sense, adapt its properties and therapeutics, and react to the bioenvironment [11]. According to [12], from the second degree, the material can be considered as “smart”. However, in many conditions, the second level of biomaterial still cannot perform the best outcome as it cannot respond to stimuli [13].

These highlight the gap between a normal biomaterial and a smart material. Smart materials can react to external and environmental stimuli [14,15]. Types of stimulation can be classified as chemical stimuli (pH, ion, gas), mechanical stimuli (stress, strain), biological stimuli (reactive oxygen species, enzymes, protein), and physical stimuli (light, temperature, magnetic field) [16]. The difference between smart materials and common biomaterials is that smart materials can react and show new functional properties. Meanwhile, common biomaterials exhibit fixed properties with additional functional groups that change their properties [17]. A conventional biomaterial exhibits mutual characteristics with a smart material in terms of biocompatibility, bioactivity, and biofunctionality, as well as biomedical applications. Nevertheless, a smart material has an intrinsic ability to respond to external stimuli and helps to tailor the biological environment [18], which has the edge over a conventional biomaterial for biomedical applications like bone regeneration [19]. Examples of biomaterials are polymers, ceramics, metals, and composites.

The development of smart materials benefits tissue engineering which offers a great opportunity in tissue regeneration, enhancing defective tissue functions or organ replacement either in vitro or in vivo. Tissue engineering aims to meet the downside of organ transplantation such as donor shortage and immunosuppression therapy during organ transplantation. Skin, cartilage, cardiovascular, neural, and bone engineering are the current types of human tissues that can be reconstructed by today’s technology.

The selection of smart polymers should be ideal for use in tissue engineering and the most important criteria to be considered include the effect on the body’s metabolic process, biodegradability, biocompatibility, immunogenicity, and the stability to avoid body repulsion. In addition, a good smart material should also be able to exhibit physical and chemical signals along with spatiotemporal accuracy which cannot be achieved using an inert biomaterial. Smart materials made of proteins have shown interesting applicability across a range of fields in tissue engineering due to their intrinsic biological compatibility, adaptability, and capacity to replicate the extracellular matrix (ECM) environment. Particular protein domains can be included to change their conformation in response to environmental inputs, which would then enable them to exhibit stimuli-responsive behaviour [20].

In the case of bone tissue engineering, bioactive peptides or growth factors that encourage osteogenesis can be included in protein-based scaffolds to imitate the natural bone microenvironment [21]. Similarly, in neural tissue engineering, protein-based smart materials can be developed to support the development of neuronal cells, promote axonal regeneration, and direct the construction of neural networks [22]. In addition to that, there are also a wide variety of tissue types that can be engineered by using different types of smart material scaffolds to achieve the best tissue regeneration results (Table 1).

The dissimilarity of a regular biomaterial and smart material is also shown in the previous work by [28]. It is indicated that the properties of a smart material, Nitinol (NiTi), are more effective to be used as a material for bone plates compared to a biometal, stainless steel. NiTi is a metal alloy composed of nickel and titanium, which can deform or return to its original shape by controlling the heat supply [29]. Because of the shape-memory effect, NiTi-based bone plates can apply sustained pressure continuously on the fractured bones that are influenced by the body temperature. In contrast, stainless-steel-based bone plates are only functional when pressure is applied manually, and that only lasts for a few days.

The key component to start tissue engineering is certainly the scaffold because it can degrade at the same rate as that of the tissue growth [26]. As defined by [30], a scaffold is a temporary 3D matrices material that mimics the extracellular matrix, able to support cells three-dimensionally to stimulate cell growth and formation of the desired tissue. In other words, the scaffold acts as a template for tissue formation by allowing cells to migrate, adhere, and differentiate as the scaffold is supplied with the growth factors and stimulation needed for cell growth [31].

Herein, different types of smart materials can be used to fabricate scaffolds for tissue engineering but in the future, tissue engineering and regenerative medicine will be significantly influenced by protein-based smart materials as techniques for protein engineering, biofabrication, and our knowledge of the interactions between cells and materials continue to progress. By providing specialised responsiveness to stimuli, greater regenerative characteristics, and improved biomedical applications, protein-based smart materials in tissue engineering have the potential to completely transform the field.

### 2.1. Piezoelectric Material

In the 19th century, piezoelectricity was discovered, and the word “piezo” means pressure in Greek. Literally, piezoelectric is a material that generates an electric charge in response to mechanical stress and changes its dimensions when an electric field is applied across the material [32]. Piezoelectric material converts electric signals to mechanical vibration energy and vice versa. When pressure or strain is applied, the material will develop electricity known as the direct effect.

Piezoelectric material is often used in tissue engineering, especially for bone and cartilage tissue engineering because the material has an electrical signal property that is compatible with bone metabolic activities [33]. The formation of a crystalline lattice structure exhibits a dipole moment in the piezoelectric material upon stress stimulation [34]. Piezoelectric material can emit an electrical signal or piezoelectricity. As defined by [35], piezoelectricity refers to a reverse process in which a contraction or elongation is created in the crystal once it is positioned in an electric field. Living body tissues possess bioelectricity such as piezoelectricity, pyroelectricity [36], and ferroelectricity [37] to regulate body functions and metabolisms.

However, when the material undergoes deformation upon subjection to an electric field, it is known as the converse effect. The energy harvesting capability of piezoelectric materials can be used as sensors and transducers via the direct effect; the converse effect can develop actuators for application [38,39]. In other words, they are the conversion of mechanical energy into electrical energy and vice versa, respectively. This material is known to regenerate bone and cartilage growth, and act as an electroactive scaffold [40] for neural tissue engineering due to its electrical properties. This can be achieved because the flow of ion-containing interstitial fluid through bone will generate stress, whereas the electric pulses can stimulate neurite directional outgrowth [41]. The common examples of piezoelectric materials used for tissue engineering are shown in Table 2 and Table 3 highlights protein-based piezoelectric materials used for tissue engineering.

The key point for piezoelectric materials to be introduced in biomedical applications is that the piezoelectric coefficients of body cells are like the material. In particular, numerous studies on piezoelectric properties have focused on collagen [48], keratin [41], glial cells [49], cellulose, chitin [23], prestin [50], actin, and myosin [51].

However, the most recent and challenging issues in terms of developing strong bio-piezoelectric materials and the realisation of their applications such as sensors, actuators, and energy harvesters are the limited patterning, control over the orientation, and polarisation directions. Recent attempts at biopiezoelectric material-based self-assembly, inkjet printing, 3D printing, and an electric-field-induced alignment approach have all been made in an effort to address these issues [52,53]. In contrast to a strong traditional inorganic piezoelectric material, the creation of a tool to analyse soft biopiezoelectric materials is also crucial for the fundamental research and practical uses of biopiezoelectric materials [53]. On the other hand, the piezoelectric biomolecules found in the human body are closely related to human health [54].

Piezoelectric biomolecules are found abundantly in nature such as chitin in the shells of crustaceans, cellulose from plant sources, viruses, DNA, amino acids, peptides, and proteins [22]. The piezoelectric properties from these natural sources arise from highly ordered, low-symmetry structures that lack an inversion centre [22].

Proteins (such as collagen, keratin, etc.) are composed primarily of amino acids, which also have structure-dependent piezoelectric characteristics [53]. Many different types of proteins can exist since there are hundreds of conceivable combinations of the 20 different amino acids [53]. Depending on its structure, each protein performs a certain function.

Proteins are also highly biocompatible, have a strong ability for selective recognition, and have easy functionalisation, especially in aqueous environments [55]. These properties give protein a great potential in bioelectronic applications. The wide variety of intrinsic electronic properties of protein biomaterials, especially their semiconductivity and ability to form field-effect devices, makes them highly applicable for a diverse range of bioelectronic functions for tissue engineering [55].

Collagen’s piezoelectric characteristics, for instance, have been demonstrated to influence bone formation, healing, and remodelling [53]. The spiral structure of collagen with three helical fibres each shows a lateral piezoresponse along the axis. In addition to that, many live tissue cells, including those in the tendon, cartilage, ligament, hair, skin, and cochlea, are made of different piezoelectric biomolecules which are expected to be directly tied to the state of human health [53]. Since biopiezoelectricity is strongly related to growth and remodelling in tissues such as bone, exploiting the piezoelectricity of protein-based materials such as collagen would aid in creating a self-healing environment for wounds.

Various other materials also (ZnO, BaTiO_3,_ and PVDF) show intrinsic piezoelectric effects. Recently, synthetic piezoelectric materials have been developed for implantation, treatment, or detection, as the materials are able to mimic the biological piezoelectric traits [56]. However, their applications are highly limited compared to protein-based piezoelectric materials because they are not biocompatible or biodegradable. Piezoelectricity can be easily found in some mammalian tissues (for example, hair, wool, horns, and hooves) [34]. This, apart from their high biocompatibility, availability, reliability, and environmental sustainability properties, makes them highly preferable for biomedical applications especially in tissue engineering [57]. Proteins are also more advantageous in bioelectronic applications for tissue engineering compared with traditionally used materials (metals, inorganic semiconductors, and synthetic polymers) due to their optical properties, antimicrobial properties, and self-healing abilities through self-assembly [55].

Proteins are also strongly considered for biopiezoelectric materials as they have promising potential for biomedical applications and green energy niches which ensures a sustainable future in medicine and the healthcare industry [58]. Biowastes such as eggshell membranes, fish scales, fish bladders, and so on are also high in protein sources [58]. Utilising the piezoelectric properties of these waste materials for tissue engineering purposes is one of the greatest sustainable invention approaches. Recently, fish-skin collagen and gelatine were used to produce biosensors in the form of e-skin which could monitor physiological signals that may provide information regarding medical conditions [58]. These sensors could also be powered by nanogenerators produced from the same materials, proving their ability to function as self-powered devices. Apart from that, lysozyme, a major globular protein in egg white and mammalian secretions, has also been found to exhibit piezoelectricity, with the longitudinal piezoelectric coefficient measured using PFM being 19.3 pm V^−1^, a relatively high value for biological material [58].

Although the conductivity of these protein-based piezoelectric biomaterials or any bio-based biomaterials is not comparable to those of highly conductive synthetic polymers and inorganic materials, more research into the charge transfer and electronic properties of proteins is being conducted [55]. Researchers have used various fabrication techniques such as electrodeposition and self-assembly to fabricate a more ordered structure of a natural piezoelectric material with an improved piezoelectric coefficient [59]. Etching, nano, or microstructural surface modification and direct chemical functionalisation are also techniques used to improve the performances of protein-based or generally any bio-based piezoelectric materials to be comparable to synthetic polymers [59]. One such example, elastin, which can be found in lungs, skin, and blood vessel walls, is also a widely studied fibrous protein for piezoelectricity. The piezoelectric coefficient of elastin has been found to be 0.1 pm V^−1^ which is lower than that of collagen [58]. Thus, researchers have used electrical poling techniques to enhance the elastin piezoelectric strength [58].

Apart from that, protein-based piezoelectric materials are also combined with other natural polymers or synthetic polymers to form composite membranes [59]. For example, the shear piezoelectric constant of collagen is around d14 = 0.1 pm V^−1^, but recent studies added chitosan as well as adjusted the pH from acidic to neutral which tends to improve this value [34].

Generally, in tissue engineering applications, the electrical character of piezoelectric materials can be used to fabricate scaffolds via its potential difference to regulate the voltage-gated ion channels, thus facilitating cell proliferation and cell differentiation [5]. The importance of a natural electrical current for cell proliferation is shown by [60], where the studies conducted also emphasise the bioelectricity that is needed for tissue regeneration. The generation of bioelectricity is primarily caused by the ion exchange that happens between the extracellular matrix and cell membrane which creates a membrane potential. The connective tissues usually responsible for this are constructed of fibrous proteins such as collagen [48], keratin [41], motor protein myosin, and associated protein actin [51].

Research by Liu et al. [61] has shown that piezoelectric composites have great compatibility with bone cells that successfully increase cell viability by culturing cells on the composite matrix. The findings have displayed the potential application of piezoelectric materials that are appropriate for other types of body tissue engineering as well. Bone and cartilage engineering is the most significant application using a piezoelectric material because both of them show a significant piezoelectric coefficient that can generate electrical signals themselves in response to mechanical signals [19]. This can be achieved due to the ability of a piezoelectric material to mimic the natural electricity in body cells. Bone is composed of a collagenous, flexible, and rigid extracellular matrix (ECM) with mineralised inorganic materials [62]. In contrast, cartilage is a malleable tissue because of its flexible ECM formed by chondrocytes and also composed of proteoglycans, different types of collagen fibres, and non-collagenous protein [63].

One of the approaches to engineer bone and cartilage tissues is by using a piezoelectric-based scaffold that mimics the ECM of the tissue. The reason why a piezoelectric-based scaffold is superior to a conventional scaffold is that the smart scaffold is responsive to environmental stimuli and can transfer the bioelectricity to target tissues [23], which a static scaffold could not achieve. Studies by [64] showed that piezoelectric scaffolds can generate charges to stimulate the differentiation of human mesenchymal stem cells (hMSCs) into chondrocytes by using a polyvinylidene fluoride-trifluoroethylene (PVDF-TrFE) fibre. At the damaged site of the tissue, the piezoelectric scaffold is implanted to replace the impaired ECM to support the cells [65]. Here, our body acts as a functional load to trigger the smart piezoelectric scaffold. The piezoelectric scaffold shows its “smartness” when it uses the functional load as stress to become stimuli and convert it into electrical signals via the piezoelectric phenomenon. The presence of electrical signals will initiate the calcium (Ca^2+^)-calmodulin signalling pathway that is responsible for regulating osteoclast activity in bone metabolism [65,66]. Cell proliferation and development will occur; thus, it will regenerate new bone tissues via osteoblast, whereas it will regenerate cartilage tissues via chondrocytes to heal the damaged site based on Wolff’s Law [67].

Furthermore, piezoelectric is a promising material that can be used for neural tissue engineering. As mentioned previously, bone and cartilage engineering require a converse piezoelectric effect, but neural engineering involves the direct piezoelectric effect [68]. By using an electric scaffold, it can communicate with the signals, thus being able to carry out new cell formation and repair on the synthetic matrix [69]. Electrical and thermal stimulation had successfully triggered the outgrowth of dorsal root ganglion on an electrospun PVDF-TrFE scaffold [70], which gave a reference on the probability for further neural applications research. Studies by [71] also showed positive results of rat spinal cord neurone regeneration by an increment of 79% neurone growth on a piezoelectric PVDF film, compared to a regular film with no piezoelectric properties. The stimuli used were external mechanical vibration stimulation to trigger a response between the scaffold and the body tissues.

Other than using a smart scaffold, piezoelectric actuator implantation showed significant results in bone regeneration [72]. The piezoelectric actuator is a transducer device that converts electrical signals into a mechanical displacement via a converse piezoelectric effect due to the applied electrical field in a small volt [73,74]. Reis et al. [72] used a piezoelectric actuator constructed via polyvinylidene fluoride (PVDF) films and silver electrodes to achieve bone-healing results. In this case, the bone regeneration was influenced by mechanical stress stimulation. Via osteotomy procedures, the PVDF film-based actuator was implanted inside the slot osteotomy cavity. The results showed an increase in the total bone area by percentage as well as osteopontin production around the actuator site compared to the negative control set. The researchers concluded that a piezoelectric material associated with an electrical current can enhance osteoblast proliferation and other mechanisms to stimulate bone growth at the film–implant interface.

Overall, protein-based piezoelectric materials are very promising candidates for future generations of “green” piezoelectric materials and piezo devices [34]. More research should be focused on improving the bioelectronic performance by designing better protein-based piezoelectric materials to overcome the current limitations.

### 2.2. Shape-Memory Material

Shape-memory material plays a dominant role in the biomedical sector, and it is notable due to its shape-memory effect character, which can change its structure upon stimuli and return to its original state via an external stimulus [75]. The shape-memory material is a novel material that has been widely used in the biomedical sector for vascular stents [76], drug delivery [77], self-tightening sutures [78], orthodontic devices [79], wound dressing [80], and more. Herein, this section will discuss the utilisation of the smart-memory effect (SME) of smart-memory materials to perform different types of tissue engineering.

Shape-memory materials can be categorised into three types, mainly shape-memory alloys (SMAs), shape-memory polymers (SMPs), and the newly discovered shape-memory hybrids (SMHs). SMAs are favourable to be applied in various fields taking into consideration their non-corrosive toughness and ductile properties. Nickel–titanium (Nitinol) (55% nickel–45% titanium alloy) is one of the most extensively used SMAs in the biomedical sector as it has high mechanical performances and biocompatibility. Among all metal-based SMAs, Nitinol has the greatest shape-memory effect and super-elasticity properties due to the transformation from the martensite to austenite phase [81].

The SME has also been defined as a physical deformation of a material when triggered via thermal stimulation above a certain temperature [82]. However, in recent studies, stimulation such as chemicals [83], light [84], and mechanical force [85] have also been able to trigger the response of the material to achieve the SME. In order to execute the SME, the shape-memory material undergoes reversible thermoelastic martensitic phase transition due to its peculiar crystalline structure [86].

The shape-memory effect can be achieved by supplying external stimuli, the temperature in the process of heating and cooling the alloy. The martensite phase is where the alloy can be deformed at low temperatures with a crystal structure and lower symmetry. At the martensite phase, shape-memory materials are soft and easy to be deformed at low temperatures. The material will return to the original/parent shape upon high heat over the reverse transformation temperature while the material has a high degree of symmetry [87]. Austenite occurs at high temperatures where the material has a crystal structure and a high degree of symmetry. In contrast, a conventional material would not have the thermoelastic and martensite transformation that gives rise to the SME [88]. There are also two types of SME, they are one-way SME and two-way SME. One-way SME shows its recovery effect only when it receives thermal stimulation, whereas two-way SME shows its recovery effect during both cooling and heating processes.

**Figure 2:**
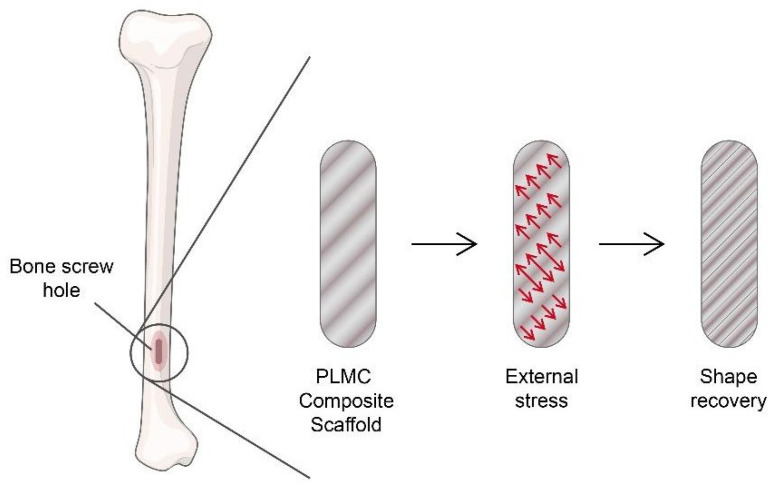
PLMC composite scaffold SME on filling bone screw hole (modified from [92] and created using Adobe Illustrator).

Undoubtedly, the scaffold is one of the common ways to engineer tissues. The SMP-based scaffold has been used to achieve the optimum efficiency in tissue engineering, especially in the neuronal, orthopaedic, and cardiovascular sectors. Bone defect occurs when one particular part of the body lacks bone tissues [89]. Studies by Zhang et al. [90] showed a self-fitting SMP scaffold fabricated by poly(e-caprolactone) (PCL) with shape-memory properties to heal cranio-maxillo facial bone defects. The PCL-based scaffold was indulged with shape-memory behaviour via a heating and cooling procedure. In the martensite-like transformation phase, an SMP will be easily deformed into any shape. So, the SMP-based scaffold allows the scaffold to fit into the defected site to enhance osteoconductivity and allow cell proliferation and osteogenic gene expression. On condition of the accomplishment of the experiment, it is possible to treat other types of bone defects. Meanwhile, the SMP of a polyurethane porous scaffold fabricated by Yu et al. [91] proved that the polyurethane-based scaffold possesses the SME, which can promote cell proliferation for bone tissue engineering, as shown in Figure 2. However, they only suggested it for bone tissue engineering, though the polyurethane scaffold could be a suitable medium for other types of tissue engineering as well.

As for neuronal engineering, an elastomeric polymer poly(octamethylene maleate (anhydride) citrate) (POMAC) SMP scaffold was fabricated to engineer cardiac patches in vivo [93]. The cardiac patch was injected into the host’s heart infraction site and stimulated via external stress to trigger the SME of the scaffold to return to its original shape. The injection was associated with minimally invasive surgery (laparoscopy) rather than an open-heart surgery to minimise the rate of complications and operative blood loss [94]. The result showed that the cardiac tissues carried by a shape-memory-based scaffold can improve left ventricular function. The conclusive research acted as a fundamental study for organ repair with other types of functional tissues, such as parenchyma cells [95,96].

As reported by Han et al. [97], a pH-sensitive SMP made by β-cyclodextrin-modified alginate (β-CD-Alg) and diethylenetriamine-modified alginate (DETA-Alg) is able to reform the original shape at pH 7 with great tensile strength, to become biodegradable and biocompatible. These findings show a prominent characteristic for tissue engineering as the recovery pH value is very close to selective internal body organs [98]. Different types of tissues can be engineered or remodelled to cooperate with the SMP therefrom. Based on the experiment by Han et al. [97], the initial shape (parent shape) of the SMP is straight. Then, we fixed the desired “U” shape at the recovery pH level, pH 7, and the SMP was immersed in a higher pH level at pH 11.5 for 2 min. When the alkalinity is removed, the SMP will remain in its “U” shape until the material meets its recovery stimulation at pH 7. At pH 7, the SMP will return to its initial shape. The same technique can be applied to this SMP according to different body parts at other pH values.

**Figure 3:**
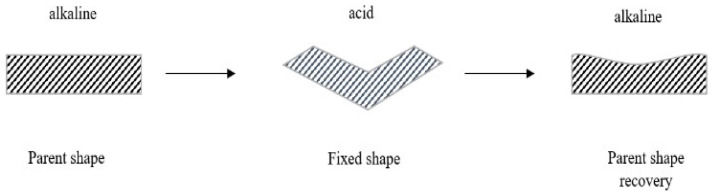
Preparation of pH-sensitive SMP with SME (created using Adobe Illustrator).

Regardless of any type of stimulation-induced shape-memory materials, they need to undergo certain processes to have SME properties. Shape-memory materials have distinctive chemical structures and molecular networks that are capable of “remembering” the programmed shape. Firstly, the parent shape is the condition of a polymer where its shape is our desired condition which the material is needed to be exposed at a certain external stimulation. Then, the same stimulation with different intensities is needed to act on the material to fix it at a temporary shape [99]. In order to recover the parent shape of the material, the exact same stimulation during the original state is acted on it. According to the experimental theory by Song et al. [100], we can deduce that the SMP has undergone alkali and acid processes to establish the pH-triggered SME that has an 80% recovery rate (Figure 3). At a higher pH (pH 9), the carboxylic dimers of the SMP are damaged. Based on the study, the lower the pH, the better performance of the recovery rate.

It is recommended to use pH 1 to pH 2 compared to pH 3 to pH 4, as the dimers will be re-built at a lower pH to fix the shape. At this stage, the desired shape is fixed by applying external force. The reformation carboxylic dimers of the parent shape will again be broken down when the recovery process takes place at the initial pH level (pH 9), which the material “remember” the original state of the shape. However, this SME only recovers 80% of the initial shape. Li et al. [101] also supported the studies, in which they explained that a pH-induced SME is aided by hydrogen-bonding interactions through changing the pH value. An abundance of hydrogen bonds is formed at a lower pH to fix the shape; the dissociation of hydrogen bonds occurs at a higher pH to deform and recover the shape. A material’s 100% shape recovery rate might be studied further. With successful SME programming, a selective pH or other stimuli can be planned according to organs that have different chemical properties. Table 4 shows other types of SMPs and their functions in tissue engineering.

Features of shape memory, although traditionally associated with synthetic materials, have also been observed in biological substrates especially in the secondary structures of protein due to their structural metastability [105]. The keratin α-helices arranged in a coiled architecture undergo a structural transition into metastable β-sheets continuously when a load is applied along their longitudinal axis [105]. This mechanism can be either reversible or irreversible depending on the α-keratin species. This mechanical transformation can be observed in biological tissues such as sea snail egg capsules, animal skin, or even certain hair species where this transformation has been selected by nature to provide protection and enable physiological functioning in response to external stress [105].

Recently, protein-based hydrogels have been found to have increased stiffness and a narrow tunability range which allows for shape-programmable behaviour [106]. Proteins comprising covalent hydrogels could be termed dynamic hydrogels mainly due to their mechanical properties [107]. The proteins can form in situ in physiological environments, are reversible under pH, and confer self-healing, shape memory, and stimuli-responsive properties to the hydrogels [107]. Easily processible proteins are excellent candidates for dynamic biomimetic hydrogels. Dynamic protein hydrogels can transform with external triggers and spatiotemporally varying cues [107]. The mechanical flexibility from conformational changes may be due to protein folding, reversible crosslinks, or both [107].

A mutually exclusive protein-based folding–unfolding switch controlled via a redox reaction exhibits a Young’s moduli between 10 and 40 kPa [107]. Such cyclically varying Young’s moduli could also reversibly direct cell spreading in human lung fibroblasts [107]. There is also another study which shows that a dynamic protein dual-network hydrogel with a breaking strain of up to 1200% could mimic the muscle structure and motion through the molecular sliding and folding–unfolding properties of protein [107].

It has been quite challenging to obtain the same smart behaviour as that of polymer-based hydrogels due to the limitation in the range of solvents, concentrations, and temperatures that can be used [106]. However, the shape-memory ability in protein-based hydrogels is limited by the minimum protein concentration required for gelation, and at the higher end by the solubility of the protein [106]. A shape-memory approach based on (un)folding transitions of protein currently does not exist [106].

### 2.3. Polymeric Hydrogels

Hydrogels are a hydrophilic three-dimensional (3D) network structure with a high capacity to hold large amounts of water or biological fluid that are prepared by either physical or chemical crosslinks [108] in the polymer network. Hydrogels upon stimulation (pH, glucose, enzymes, magnetic field, temperature, light, et cetera) will react due to their swelling and deswelling behaviour, change in hydrogel volume, water absorption capacity, sol–gel phase transition, conformation, and solubility [109].

A physical crosslink hydrogel network exhibits weak bond interactions as it is prepared via molecular entanglements such as hydrophobic interaction, ionic interaction, hydrogen bonding, and crystalline formation. Meanwhile, a chemical crosslinked hydrogel possesses a stronger covalent bond between polymer chains, including methods of molecular crosslinking, hybrid polymerisation, photosensitive agents, and enzymatic crosslinking [110,111]. Due to the strength of bonds, the gel formation of physical hydrogels is reversible but less stable whereas a chemical hydrogel is irreversible but very stable [110].

Hydrogels are increasingly being used in pharmaceuticals, biomedicine, and bioengineering due to their simple fabrication techniques and great sensitivity to environmental stimuli. Hydrogels can be derived from natural, synthetic, or semi-synthetic sources. The first ever polymeric hydrogels are poly(2-hydroxyethyl methacrylate) (pHEMA), developed by Wichterle and Lim for contact lens bio-application [112]. Hydrogel is a prime selection of material because it is compatible with living tissues, and also emulates the extracellular matrix structure necessary for cell growth [113]. In addition, hydrogels show self-healing properties through dynamic covalent bonds and non-covalent interactions, beneficial for therapies and drug delivery systems [114,115].

Hydrogels are hydrophilic biopolymers that have swelling properties to cause volume change upon environmental stimuli, which makes the hydrogel “smart” [116]. They also exhibit different behaviours in response to stimuli. They can swell [117], undergo sol–gel transition [118], change in network structure [119], permeability [120], or mechanical strength [121]. The stimulation could be light, pH, temperature, stress, magnetic field, glucose, radiation, et cetera.

Since hydrogel has a high ability to retain water, its physical properties are close to soft tissues to be constructed as a 3D scaffold for tissue engineering [122]. The advantages of synthetic polymeric hydrogels over natural hydrogels are the self-adjustment of the hydrogel molecular weight, mechanical strength, and biodegradability [122]. Due to the swelling behaviour of hydrogels, researchers have found that polyvinyl alcohol (PVA) hydrogels can act as a replacement for the nucleus pulposus [123]. The polymeric hydrogels are homogeneous to the swelling pressure of the nucleus pulposus once implanted in the intervertebral discs in vivo to promote tissue regeneration. Indeed, the replacement is effective, but in a long-term condition, the mechanical load stress and effects are yet to be discovered.

Magnetic-responsive hydrogels fabricated by PVA have also been developed to control the protein adsorption of hydrogels [124]. The experiment demonstrated that the cyclic variation in the magnetic field intensity (0.45 T) had a reduced protein adsorption while stimulating protein release from the hydrogel. This remarkable study indicates the opportunity to utilise the magnetic-responsive PVA for tissue engineering that is required to elevate biocompatibility and cell growth [125].

Recently, protein-based hydrogels have been gaining much attention from researchers mainly because of their functions which include fundamental building blocks, such as block copolypeptides, recombinant segments of naturally occurring structural proteins, like elastin, tandemly repeated silk-like and elastin-like peptide blocks, and recombinant triblock copolymers of a random polypeptide sequence flanked by two coiled-coil blocks and protein/peptide crosslinkers, such as intact native proteins or oligopeptides, oligodeoxyribonucleotides, stereospecific D,L-lactic acid oligomers, or by antigen–antibody interaction [7,126]. Hydrogels can also be categorised based on the source of protein, secondary structural elements, methods of assembly, and routes of delivery [127]. Classification of hydrogels into fibrous or micellar-based hydrogels is also determined via the self-assembly of proteins into fibres and micelles [127].

A smart hybrid hydrogel containing synthetic polymer-based primary chains could be imposed with a well-defined coiled-coil protein motif [7]. Proteins can also be excellent candidates when designing smart actuators responsive to temperature, ions, or electricity, considering their natural advantages especially in terms of biocompatibility, degradability, controllable modulation on specific mechanical properties, and multi-responsiveness [128]. Thus, protein-based hydrogels can not only be used for actuators, but also be designed as biosensors for glucose, proteins, bacteria, etc. [7]. For example, a highly selective glucose-sensing protein photonic crystal hydrogel was fabricated from a genetically engineered *E. coli* glucose/galactose-binding protein [128].

In general, protein-based hydrogel sensing systems have advantages such as enhancement capacity for target analytes, thus improving the sensitivity, facile modulation of the composition of sensing molecules, minimising the autofluorescence/background of plastic sensor devices, offering a highly aqueous atmosphere that is biologically compatible, and preventing non-specific protein adsorption, which provides more stimulus for the development of this field [7].

In addition to that, protein-based hydrogels are being engineered through techniques such as moulding and photoactivated patterning to mimic the dynamic 3D microenvironment of the extracellular matrix (ECM) for better application in tissue engineering [127,129]. Hydrogels formed using fibrous proteins (e.g., fibrin, keratin, collagen, elastin, and fibroin) can simulate ECM-like porous networks, encapsulate cells, and facilitate cell adhesion, proliferation, metabolism, and differentiation [107]. Table 5 shows some examples of protein-based hydrogels with their composition, properties, and application in tissue engineering.

The use of exosomes, which are the small extracellular vesicles that are involved in cell-to-cell communication, has been recently recognised especially in therapeutic applications. The functional proteins, nucleic acids, lipids, and small exosomes act as great delivery vehicles. However, there is a critical need to formulate better strategies especially to increase their retention time as they have a rapid clearance rate from targeted organs [127]. Recent studies have used silk fibroin hydrogels to deliver microRNA (miRNA) packaged into exosomes derived from stem cells to prevent vascular ageing [127]. Peptide-based and protein-based hydrogels show a promising way of delivering exosomes in the near future. Moreover, one of the first self-healing, fibrin-based injectable hydrogels that could promote the formation of new blood vessels (angiogenesis) and restore blood supply to blocked vessels via injection alone has been recently designed using chitosan [107].

Hydrogel scaffolds should ideally stimulate cell differentiation, be less cytotoxic to healthy cells, exhibit high compressive strength, promote good cell survival, and possess dynamic reactivity to physiological stimuli to be applicable in tissue engineering and regenerative medicine [107]. Biomedical hydrogels often require diverse properties blended in one system, such as structural memory, quick self-healing ability, high dynamic mechanical resilience, and excellent physiological stability in order to function properly [107]. Protein hydrogels are largely being used for in vivo regenerative implants as well as to repair, replace, develop, and support tissues because they have very favourable properties [107].

Although natural hydrogels have more advantages than synthetic hydrogels, synthetic hydrogels (polymeric hydrogels) have better mechanical properties which can customise the composition, modify the firmness, and are easy to fabricate [133]. Synthetic hydrogels can be classified into homopolymeric, copolymeric, and Interpenetrating polymer networks (IPNs) that have different molecular structures (Table 6).

However, researchers are working on producing dynamic protein hydrogels with autonomous assembly, structural memory, quick and high self-healing ability, high mechanical resilience, cell affinity, tissue adhesiveness, toughness, and excellent physiological stability integrated in one system which would be highly useful and promising in the future biomedical industry [107].

Furthermore, hybrid poly(N-isopropylacrylamide) (PNIPAAm) hydrogels with a PCL scaffold tend to encourage chondrogenesis [137] along with thermo-sensitive properties. The results showed that the scaffold application was able to increase human mesenchymal stem cells [138]. The temperature acts as an external stimulus which activated the sol–gel transition, which then allowed the cells to be encapsulated in the hydrogel. Literally, the hydrogel sol–gel transition means the change in the state of a fluid into gel form, and this reaction is reversible. When PNIPAAm is below the lower critical solution temperature (LCST) of 32 °C, the dissolution of the polymer polar groups and water molecules bond occurs; when it reacts upon high heat in water, hydrophobic interaction occurs and the polymer precipitates [118]. The precipitation state is where the polymeric hydrogel retains its shape without external support to be used as a stable scaffold.

**Figure 4:**
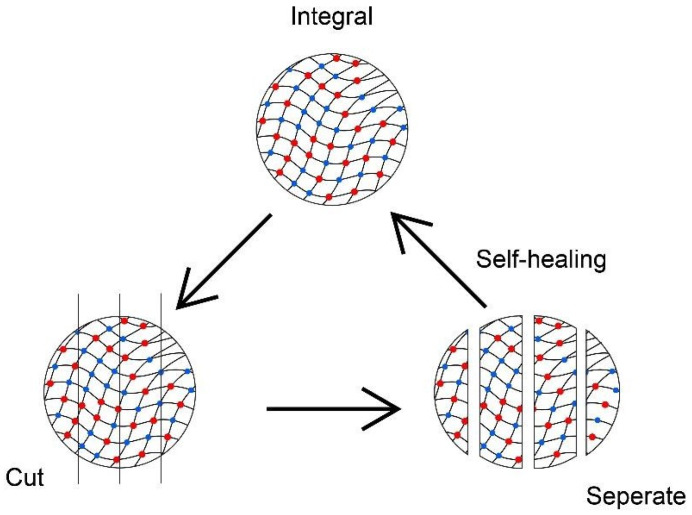
Self-healing hydrogels process happening in circular hydrogels which has been cut into several pieces (created using Adobe Illustrator).

The reversible sol–gel transition in addition to self-healing properties can also be influenced by the pH changes. Polymeric hydrogel poly(triazole) (PTA) had shown its redox property to carry on the sol–gel transition and self-healing efficiency of 70.6% without the presence of a catalyst [139]. The self-healing behaviour is aided by the acylhydrazone exchange reaction, especially at an acidic condition of pH 4.5 [140], whereas the disulphide exchange involves the homolytic cleavage of the disulfide bond and sulphur-based radical transfer to express the self-healing behaviour [141]. When the hydrogels are separated into two sides, the acylhydrazone and disulfide bond reconstruct and consequently re-joint the hydrogels into their original state. Figure 4 shows that the cut hydrogel is separated, then cohered into one piece within 24 h. The healed hydrogel is still able to withstand tensile force, such as stretching and compressing. These cutting and self-healing processes can be repeated a few times without weakening its physical and mechanical properties.

A self-healing hydrogel could benefit tissue engineering. Tseng et al. [102] reported hydrogels with self-healing properties are favourable to regenerating neural stem cells after a few cycles of intentional high-strain destruction. Self-healing properties of hydrogels are also prominent in tissue complex regeneration, as shown in a study by Hou et al. [142]. The integration of chondrocyte-encapsulated hydrogels as a scaffold with bone was developed to achieve cartilage–bone tissue complex regeneration. The complex model showed the prospect of reconstructing other types of body tissue complexes to restore tissue caused by congenital disorders or injuries.

## 3. Advantages of Protein-Based Smart Materials in Tissue Engineering

Smart material is a wise clinical invention in the regenerative biomedicine field due to its high-value application in tissue engineering. There are a few main emphases of the biomaterials that are ready to implant in vivo. The biomaterial should be biocompatible, biodegradable, biofunctional, and bioactive to native body cells. This paper focuses on protein-based smart materials and it is more challenging for man-made materials to have those ideal characteristics that are necessary to pair seamlessly with body tissues.

Polymers have wide applications in the biomedical sector due to their biocompatibility. Biomedical polymers can be classified into natural polymers and synthetic polymers. Synthetic polymers have become popular in recent years due to their ability to be tailored to different products or applications and functions according to demand, especially stimuli-responsiveness [143]. A low toxicity, biocompatibility, biodegradability, high tensile strength, and biostability are examples of properties that can be fabricated by man. The preponderance of synthetic polymers is also highlighted because the mechanical properties and degradation rate of natural polymers are hard to control unlike the synthetic ones [144]. However, biocompatible but non-degradable SMPs are still in use for surgical procedures for repairing and supporting purposes. Infusing biomaterials for temporary support until the tissues are fully recovered may burden the patient’s physical and mental health endurance as the material will remain in the body and would require a second operation to remove it [145].

On the other hand, protein-based materials are highly promising since they can be altered or modified in terms of both their shape and chemical makeup [8]. Researchers are able to create a protein form that is best suited for a particular application due to the variety of ways in which the raw protein of these materials is processed [8]. For example, protein can be treated so that its malleability and softness mimic circumstances that could promote cell development, enhancing the biocompatibility of these materials and the range of their applications [8]. Additionally, the intrinsic biodegradability of these materials and their role in a circular economy, which transforms waste products into beneficial new materials, are of incalculable significance [146].

Protein-based materials are advancing quickly to soon become the prominent options for problems of biomedical engineering, materials science, waste management, and beyond [8]. The incorporation of genetically engineered proteins with ligand binding and allosteric sites into hydrogel networks generally produced a swelling response that could be fine-tuned over the regulation of hydrogel behaviour [7]. This illustration demonstrates that proteins and peptides have well-defined and homogenous structures, consistent mechanical characteristics, and coordinated folding/unfolding transitions when compared to synthetic polymers [7]. Thus, the coupling of peptide/protein domains with synthetic polymers may result in unique materials with properties that are better than those of the constituent parts separately [8].

Protein-based products are extremely desirable since they are accessible, a part of a circular economy of source materials, biocompatible, and biodegradable. Furthermore, many structural proteins are very modular and simple to manipulate due to the fact that they are polymeric materials made up of discrete repeats of amino acid sequences [147]. A particular amino acid sequence that is found to impart desirable properties can frequently be fused with another protein, combining the desirable properties of the two [8]. For instance, fusing a sequence that gives a protein its strength with a sequence that gives it its elasticity can result in a material that is both strong and stretchy [8].

Natural proteins including silk, elastin, resilin, collagen, and keratin are among the strongest and most elastic substances known to man [148]. The idea of combining multiple proteins to create a substance that possesses the advantageous traits of both is another area of protein-based materials that is currently being researched. The silk-elastin-like peptides (SELPs), which have been electrospun and utilised to make hydrogels and nanoparticles for drug administration, as well as biocompatible fibrous scaffolds with applications in tissue engineering, are one particularly remarkable example [8,148]. However, globular proteins’ capacity to produce novel materials is also starting to be explored. These proteins have peculiar physical properties [8,148]. One example is the bacterial hydrophobin BslA, which forms strong monolayers by self-assembling at hydrophobic/hydrophilic interfaces. Medication distribution is made possible by its ability to stabilise emulsion droplets, and it may even improve medication absorption [149,150].

In addition, despite a natural-derived hydrogel being biocompatible to natural tissues, it is low in mechanical strength which restricts its potential to execute tissue engineering [151]. Hydrogel carries a high content of water of more than 97%, resulting in a weak component makeup, which means it is easier to break [152], which makes it a disadvantage for effective implantation. However, a smart hydrogel with pressure-sensitive properties is good for overcoming the limitation. A nanofibrous hydrogel can sense a small indefinite quantity of pressure (>50 Pa), along with a high durability which can withstand above 100 cycles of compression [153]. The robust and pressure-responsive hydrogels are competent to benefit numerous types of tissue engineering.

A smart hydrogel can change its volume in response to stimuli such as pH, electrical field, temperature, and light. Hydrogel PNIPAAM and poly(N-vinylcaprolactam) (PNVCL) show their smart character influenced by thermal changes by the coil to globule transition of the network arrangement from soluble to non-soluble in water [16,154,155]. Even a minute change in thermal fluctuation can trigger the swelling–deswelling transition mechanism [156]. This smart response of polymeric hydrogels is capable of being utilised as a material for scaffold fabrication. As reported by Zhang et al. [157], a thermo-responsive PNIPAAM-based hydrogel performed well for biocompatibility for osteochondral regeneration and chondrogenesis-related genes were effectively expressed. Therefore, more research should be conducted in order to completely utilise protein to be incorporated into smart materials for tissue engineering to reap more benefits.

## 4. Future Prospects of Smart Materials in Tissue Engineering

Despite the techniques of tissue engineering being more advanced compared to the past, there are still many limitations and challenges that are pending to be solved. For instance, scaffolding for cardiac tissue engineering is not ideal yet because it is difficult to imitate the anisotropic structure of human heart tissue [158]. Nonetheless, the immune rejection of foreign object insertion remains a doubt. The body’s immune system will fuse and accept the biomaterial with an improvement in the way of fabrication when there is further comprehension of the interaction between substances and immunity mechanisms.

The advancement of smart materials will also lift their sensitivity and precision towards stimuli by increasing the accuracy statement of units, such as the pH, temperature, and electromagnetic waves. Furthermore, the better mechanical strength of hydrogels will be developed for scaffolding in tissue repair. In addition to application in tissue engineering, the application in drug delivery, medical devices, wound management, etc., will be benefited as well. In the future, the upgraded smart materials will be ready to meet newly emerging diseases and health afflictions.

Not to be neglected is that tissue engineering has often been a challenge for paediatric patients. However, most of the research and development focus has been on mature adults and we use them as a first interest for analytical study. Children should be actively involved in clinical trials for healthcare improvement [159]. In particular, bone regeneration is a great accomplishment in adults, but the immature tissues and continuous growth in children have been an issue for tissue engineering implantation [160]. Since the organ systems of children are not fully developed yet, surgery for tissue engineering implants poses a certain degree of risk for them. In relation to this, researchers are recommended to give more attention to paediatric tissue engineering by using the advantage of smart materials.

Smart material implementation is growing rapidly in all sectors, not only in the healthcare sector. Even so, the growth and demand for smart materials are hindered by the high cost and availability. Proteins and assemblies have been used in a bottom-up manner, in homogeneous or heterogeneous settings, or via physical incorporation to create “smart” materials [7]. However, there are several difficulties in this area. Since large-scale manufacture is not yet possible for the actuators, more research with accessible proteins should be conducted [7]. Artificial tissues have already been created for protocell models, but when it comes to functionality, there is still a significant gap between artificial protein models and genuine tissues [7]. So, it would be beneficial to look more closely at how these models compare in terms of their composition, building blocks, and properties. It is also crucial to enhance the fundamental requirements of sensitivity, stability, and reproducibility.

Further research into the roles and applications of enzymes is necessary because there are numerous ones in biological systems whose activities are yet unknown [7]. Peptide- and protein-based biomaterials still need to overcome some fundamental limitations for practical applications, especially in tissue engineering. Firstly, the current research is very much focused on a small number of peptides and proteins while there is a massive library of potential natural and synthetic peptides and proteins that should be researched [86].

Additionally, both experimental and theoretical approaches should be used to thoroughly investigate the underlying causes of their electrical characteristics. The performance of protein-based biomaterials also needs to be improved rather than just focussing on the behavioural properties [86]. For instance, the processes of self-assembly during the manufacture of the conducting materials or devices and large-scale production for piezoelectric energy harvesters should be focused on as well [86]. Due to insufficient research, it is currently challenging to create high-performance bioelectronic materials that are affordable and have controllable features. In addition to that, proteins have stability problems due to their molecular and physical characteristics, so it is important to take their physical and chemical stability into account [86]. For example, changes in the temperature and pH frequently impair the performance of peptides and proteins. The goal of enhanced green bioelectronics can be achieved by developing new biocompatible and multifunctional devices using machine learning and artificial intelligence [86]. More detailed explorations into protein-based smart materials are required to achieve the goal of advanced green bioelectronics.

We expect more research and development will be conducted to master smart materials in order to pursue further remodelling and manufacturing methods. In a few years’ time, it is believed that researchers will provide insightful comprehension of current smart materials at an atomic and molecular level.

## 5. Conclusions and Way Forward

This review demonstrated protein-based smart materials in the biomedical sector in regenerative medicine applications. Generally, smart biomaterials can react upon environmental changes and stimuli to accomplish various types of tissue engineering, from cardiac to orthopaedic cases. They can give reactions in response to chemical, mechanical, and physical stimuli customised for distinct tissue engineering. Smart biomaterials have a proven great biocompatibility, biodegradability, and biofunctionality to be transplanted or substituted in our bodies. This paper also focused on the advantages of protein-based smart materials over conventional or traditional biomaterials. Protein-based smart materials have promising characteristics to be used as fundamental materials to reconstruct tissue. There are potentialities of the material to be further explored to have a better performance in terms of sensibility. The research and development of protein-based smart materials are crucial approaches to further explore the potential application of smart materials till clinical application.

## Figures and Tables

**Table 1:** Types of scaffold material used for tissue engineering.

Tissue Engineering	Scaffold Material	Effect	References
Neural tissue	**Piezoelectric polymers**: Polyvinylidene fluoride (PVDF) Poly[(vinylidene fluoride-co- trifluoroethylene] (PVDF-TrFE) Poly(3,4ethylenedioxythiophene) (PEDOT) Polylactic acid (PLLA) Poly(3-hydroxybutyrate-co-3- hydroxyvalerate) (PHBV)	Generate electrical signals	[23]
Cardiovascular tissue	**Polymeric scaffold**: Polyethylene terephthalate (PET) Polytetrafluoroethylene (ePTFE) Polyurethanes (PU) Polyglycolic acid (PGA) Polyesters Poly(L-lactic acid) (PLA) Poly(ε-caprolactone) (PCL) Polyhydroxyalkanoates (PHA) Polyglycerolsebacate (PGS)	Customisable material properties	[24]
Bone tissue	**Polymeric hydrogel**: Polyethylene glycol (PEG)	Release bone morphgenetic protein-2 (rhBMP-2)	[25]
Skin tissue	**Polymeric scaffold**: PCL Poly(lactide-co-glycolide) (PLGA) Polyethylene oxides (PEO) Polylactide (PLA)	Stabilise growth factor	[26]
Skeletal muscle tissue	**Shape-memory polymers**: PLA	Proliferate and differentiate C2C12 myoblast cells	[27]
Bone tissue	**Piezoelectric polymers**: (Protein) Collagen	Bone repair and regeneration	[5]

**Table 2:** Common examples of piezoelectric material and its function in tissue engineering.

Types of Piezoelectric Material	Stimulation	Functions	References
Poly(vinylidenedifluoride- trifluoroethylene) (P(VDF- TrFE))	Ultrasound	Promote cell osteogenic differentiation and proliferation, secrete ECM proteins	[42]
Boron nitride nanotube (BNNT)	Ultrasound	Stimulate axonal regeneration, promote neuronal electrical activity	[43]
PVDF nanofibrous scaffolds	Electricity	Promote unaided electromechanical stimulation on osteoblasts	[44]
PVDF-polycaprolactones (PCL) PVDF-multi-walled carbonnanotubes (MWCNT)	Electrical field/mechanicalforce	Heal wound, regenerate bone	[45]

**Table 3:** Examples of protein-based piezoelectric material and its function in tissue engineering.

Protein-Based Piezoelectric Material	Stimulation	Functions	References
Collagen	Electricity	Bone repair and regeneration	[5,46]
Silk fibroin	Mechanical force/electricity	Promotes cell growth, proliferation, and tissue regeneration	[47]

**Table 4:** Types of shape-memory material and their functions in tissue engineering.

Types of Shape-Memory Material	Stimulation	Functions	References
Thermoplastic polyurethane (TPU)	Temperature	Control the behaviour of viable stem cells	[102]
Star-shaped polylactide (PLA) with aniline trimer (AT)	Electrical field	Promote C2C12 cell adhesion and proliferation, increase osteogenic differentiation of C2C12 myoblast cells	[27]
Poly(PCL/PDMS urethane)/carbon black nanofibres	Electrical field	Promote neuronal cell proliferation	[103]
Polycaprolactone dimethacrylate (PCLDMA)	Infra-red irradiation/magnetic field	Promote NIH3T3 cells proliferation	[104]
Keratin (protein)	Mechanical field	Protects and enables physiological functioning	[105]

**Table 5:** Summarises the properties and applications of some fibrous protein hydrogels.

Hydrogel Composition	Applications	Properties	References
Collagen (Collagen/dialdehyde guar gum, guar gum/borax)	Skin wound repair	T_gel_ = 25 °C Max G’ = 1.6 kPa	[130]
Gelatin (Sodium alginate dialdehyde/gelatin (15ADA20G)	Knee injury repair	T_gel_ = 37 °C Min *t*_gel_ < 4 min Compressive strength = 295 ± 32 kPa	[131]
Gelatin (5.6% *w*/*w* oxidised starch/gelatin)	Wound healing	T_gel_ = 50 °C Elastic modulus = 36.6 kPa Compressive strength = 14.3 kPa	[132]

**Table 6:** Classification of stimuli-responsive synthetic hydrogels.

Type of Hydrogels/Examples	Composition	References
**Homopolymeric** pHEMA 2-Hydroxyethyl methacrylate (HEMA) Polyethylene glycol (PEG)	Comprised of polymer network derived from one type of species monomer	[134]
**Copolymeric** Methacrylic acid (MAA) Poly(ethylene glycol) methacrylate (PEG-PEGMA) Carboxymethyl cellulose (CMC) Polyvinylpyrrolidone (PVP)	Comprised of two or more different monomer species with at least one hydrophilic component	[135]
**Interpenetrating polymer network (IPN)** Poly(*N*-isopropylacrylamide) (PNIPAAM)	Comprised of more than one network that is at least partially interlaced on a molecular scale but not covalently bonded to each other	[136]

## Data Availability

The data presented in this study are openly available.
